# Drug-related problems and potential contributing factors in the management of deep vein thrombosis

**DOI:** 10.1186/s12878-016-0043-y

**Published:** 2016-02-04

**Authors:** Fekede Bekele Daba, Fisihatsion Tadesse, Ephrem Engidawork

**Affiliations:** Department of Pharmacy, College of Health Sciences, Jimma University, P.O. Box 378, Jimma, Ethiopia; Department of Internal Medicine, Tikur Anbessa specialized hospital, Addis Ababa University, Addis Ababa, Ethiopia; Department of Pharmacology and Clinical Pharmacy, School of Pharmacy, College of Health Sciences, Addis Ababa University, P.O. Box 1176, Addis Ababa, Ethiopia

**Keywords:** Deep vein thrombosis, Drug-related problems, Warfarin, International Normalized Ratio, Tikur Anbessa Specialized Hospital

## Abstract

**Background:**

Patients receiving anticoagulant drugs must be carefully screened for drug-related problems, as such medications, including warfarin have narrow therapeutic ranges and a high potential for complications. Thus, this study was designed to assess drug-related problems in the management of patients with deep vein thrombosis at Tikur Anbessa Specialized Hospital.

**Methods:**

A cross-sectional descriptive study involving retrospective chart review of adult patients with deep vein thrombosis was conducted from patients who visited the hospital from July 2012 to June 2013, using structured data collection format and this was complemented by key informant interview.

**Results:**

The study included 91 patients with venous thromboembolism. Fifty three (58.2 %) were females. Mean age was 38.6 (±13.76) years and more than 2/3 were below the age of 44 years. About 54 % of them presented with concurrent medical conditions and most commonly with cancer. Adjustment of warfarin dose up or down was done in increments of 16 to 100 % for recent subtherapeutic International Normalized Ratios, 16 to 50 % for therapeutic and 11 to 66 % for overtherapeutic International Normalized Ratios, with the mean of 36.5 (±18.03) based on the cumulative weekly dose of warfarin. There was significant linear relationship between percentage of dose change and consequent International Normalized Ratio values (R^2^ = 0.419; *p* = 0.000). Accordingly, more than 51 % of them presented with nontherapeutic International Normalized Ratio ranges following dose adjustment.

**Conclusions:**

The most prevalent anticoagulation drug-related problems were subtherapeutic doses, overtherapeutic doses and potential drug interactions. Institutional validated decision support tools for dosing decisions during maintenance anticoagulation therapy should be developed and used accordingly in order to prevent recurrent and hemorrhagic complications and to improve clinical outcomes.

## Background

A drug-related problem (DRP) is defined as an event or circumstance involving drug therapy that actually or potentially interferes with desired health outcomes. Categories of DRPs include unnecessary drug therapy, the needs for additional drug therapy, ineffective drug, dosage too low, dosage too high, adverse drug reaction (ADR) and patient noncompliance to the treatment [1].

DRPs are frequent and may result in reduced quality of life, and even morbidity and mortality. Despite excellent benefits and safety profile of most medications, DRPs pose a significant risk to patients, which adversely affect quality of life, increase hospitalization and overall health care cost. DRPs may arise at all stages of the medication process from prescription to follow-up of treatment [2].

Deep vein thrombosis (DVT) is the development of single or multiple blood clots within the deep veins of the extremities or pelvis, usually accompanied by inflammation of the vessel wall. The major clinical consequence is embolization, usually to the lung [3]. Acquired risk factors for thrombosis include a prior thrombotic event, recent major surgery, presence of a central venous catheter, trauma, immobilization, malignancy, pregnancy, use of oral contraceptives, myeloproliferative disorders, and antiphospholipid syndrome.

It has long been known that hypercoagulability, stasis of blood flow, and venous endothelial injury, collectively known as the “Virchow’s Triad” of pro-coagulant risk, are major factors in the pathophysiology of DVT. More recently, it has become apparent that susceptibility to venous disease is also governed by a complex interplay of gene expression, inflammation, lipid biology, and other processes. Because these processes remain incompletely characterized, standard treatment for DVT remains focused upon reducing recurrent events via the use of anticoagulant drugs.

Patients with venous thromboembolism (VTE) are generally managed with anticoagulant therapy with the aim of treating the acute event and preventing death due to pulmonary embolism (PE), in addition to minimizing the risk of postphlebitic symptoms and recurrent VTE. For most patient groups, initial therapy consists of administration of a parenteral anticoagulant drug with subsequent transition to long-term therapy with an oral vitamin K antagonist (VKA) such as warfarin for at least 3 months. Determination of the appropriate warfarin dose during initiation and maintenance therapy requires an understanding of patient factors that influence dose response: age, body weight, nutritional status, acute and chronic disease states, and changes in concomitant drug therapy and diet [4].

The goal with warfarin therapy is to maintain a balance between prevention of recurrence and excessive bleeding. This balance requires careful monitoring, typically by prothrombin time (PT)/International Normalized Ratio (INR). INR is only applicable for those taking warfarin and an INR range of 2.0 to 3.0 should be taken as therapeutic range with 2.5 as a target value for patients with DVT. INR is, therefore, used to adjust a patient’s drug dosage to get the PT into the desired therapeutic range that is right for the patient and their condition. When INR is non-therapeutic, there are many options for dose adjustments. Patients whose INR is just outside the therapeutic range can be managed by either adjusting the dose up or down in increments of 5 to 20 % based on the cumulative weekly dose of warfarin or by more frequent monitoring, the latter with the expectation that the INR will return to therapeutic levels without a dosage change [5].

Patients receiving anticoagulant drugs such as DVT patients must be carefully screened for DRPs. While receiving anticoagulants, patients must be monitored closely to ensure effectiveness and to prevent side effects or overdosing. Hence, it has narrow therapeutic ranges and is associated with a high rate of DRPs thus failing to monitor warfarin therapy could increase the risk of recurrent thrombosis and hemorrhagic complications. Therefore, this study was initiated to address the possible DRPs that could occur in the management of patients with DVT in the study area.

## Methods

The study was carried out at Tikur Anbessa Specialized Hospital (TASH), Ethiopia’s largest general Public University Hospital. A cross-sectional descriptive study involving retrospective chart review of patients with objectively diagnosed VTE and key informant interview were carried out. Data of study population were abstracted from medical record charts of patients with objectively diagnosed VTE who visited the hospital from July 2012 to June 2013, using structured data collection format. Data abstracted included the following fields: patients’ demographics, dosage schedule of the objective medications, type and number of concomitantly prescribed medications, number and types of concurrent medical conditions, series of patients’ INR values and others related conditions. For the key informant interview, 3 physicians (2 hematologists and 1 consultant internist) who had long experience in the management of DVT and were working in the Hematology unit of TASH were interviewed. The data obtained from the interview included: the treatment guideline on which current DVT management depends in the study setting, factors need to be considered in deciding dose adjustments for a given nontherapeutic INR values; instructions regarding foods rich in vitamin K intake while patients are on anticoagulation therapy and other related factors.

### Statistical analysis

Data entering and analysis were performed using SPSS software version 16.0. Descriptive statistics was calculated for demographic and clinical characteristics of patients. The percentage of warfarin dose change was calculated by dividing the difference of the total weekly dose before and after the time of adjustment by the total weekly dose before the time of adjustment and multiplying the quotient by 100 %. The association of the dosage change and the consequent change in INR value was done by simple linear regression analysis and the coefficient of determination (*r*^*2*^ value) was taken to determine the relationship between the variables at 95 % CI and p-value < 0.05 was taken as statistically significant association. Cross-tabulation was performed for a 2x2 variables and Chi-square statistic (*χ*^2^) was calculated to show the association between the variables at 95 % CI.

### Ethical clearance

Prior to data collection, the study proposal was approved by the Ethical Review Board (ERB) of School of Pharmacy as well as Department of Internal Medicine of TASH, Addis Ababa University. During data collection, name of the patients was excluded and record card numbers were used. Data analysis was performed using a code number that had been given to each patient data collection instrument. Hence, confidentiality of all information obtained from the patient’s medical record cards were kept and respected. Before conducting the interview, informed consent was obtained from the physicians.

## Results

The study included 91 patients: 87.9 % with DVT, 4.4 % with PE, and 7.7 % with combined PE and DVT. Fifty three (58.2 %) were females. Mean age was 38.6 years (±13.76 years) and the age ranged from 16 to 70 years and more than 2/3 of the study population (69.2 %) was below age of 44 years. The most common concurrent medical condition was cancer. Thirty eight (41.8 %) patients were prescribed with warfarin and other medications concurrently that might potentially alter INR (Table 1).

**Table 1:** Demographic and clinical characteristics of patients with DVT (*n* = 91)

Patient characteristics		*n*	(%)
Sex	Female	53	(58.2)
	Male	38	(41.8)
Age (years)	16–22	4	(4.4)
	23–29	25	(27.5)
	30–36	23	(25.3)
	37–43	11	(12.1)
	44–50	7	(7.7)
	51–57	10	(11.0)
	58–64	3	(3.3)
	65–71	8	(8.8)
Co-morbidities	Cancer	14	(15.4)
	HIV/AIDS^a^	10	(11.0)
	Hypertension	7	(7.7)
	Anemia	6	(6.6)
	Others^c^	16	(17.6)
Number of prescribed drugs/patient	Less than 3	55	(60.4)
	Greater or equal to 3	36	(39.6)
Number of patients with concomitant drugs (*n* = 38)	Drugs that ↑INR^b^	21	(55.3)
	Drugs that ↓INR	13	(34.2)
	Drugs that ↑ or ↓INR	4	(10.5)

^a^HIV/AIDS: Human Immunodeficiency Virus/Acquired Immunodeficiency Syndrome

^b^INR: International normalized ratio

^c^Others include venous insufficiency, pulmonary tuberculosis, heart failure, diabetes mellitus, and dyslipidemia

### Warfarin dose and INR

**Fig. 1:**
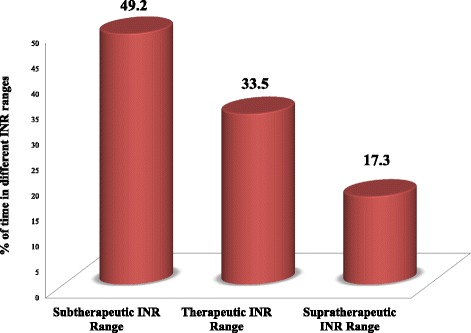
Time spent in different International Normalized Ratio ranges in patients on warfarin therapy. A series of all recorded International Normalized Ratio (INR) values were collected over 12 months for each patient and calculated to determine whether they are in therapeutic range or not while on anticoagulation therapy

A total of 415 INRs were recorded over 12 months and “percent of measured INRs in range” was used to obtain the time in therapeutic range. Patients spent 49.2 %, 33.5 % & 17.3 % of time in subtherapeutic, therapeutic & supratherapeutic INR ranges, respectively (Fig. 1).

More than 66 % (276/415) of INR values were nontherapeutic and appropriate for warfarin dose adjustment, although clinical judgment might also be considered. Accordingly, the average daily dose of warfarin was increased, unchanged and decreased for 95 (46.6 %), 102 (50 %) and 7 (3.4 %) of subtherapeutic INR values, respectively. The average daily dose of warfarin was increased, unchanged and decreased for 3 (4.2 %), 25 (34.7 %) and 44 (61.1 %) for supratherapeutic INR ranges, respectively (Table 2).

**Table 2:** Nontherapeutic INRs and warfarin dose adjustment

Nontherapeutic INRs	Decreased *n* (%)	Unchanged *n* (%)	Increased *n* (%)	Total *n* (%)
INR < 2	7 (3.4)	102 (50)	95 (46.6)	204 (100)
INR > 3	44 (61.1)	25 (34.7)	3 (4.2)	72 (100)

The daily maintenance dose of warfarin before and after adjustment differed greatly between individuals; it was commonly between 1.25 mg/day and 12.5 mg/day during both periods. The average maintenance dose during the specified period was about 5.7 mg/day before and 5.4 mg/day after dose adjustment. The dose was lower in the elderly, which was 4.2 mg/day before and 2.5 mg/day after dosage change.

**Fig. 2:**
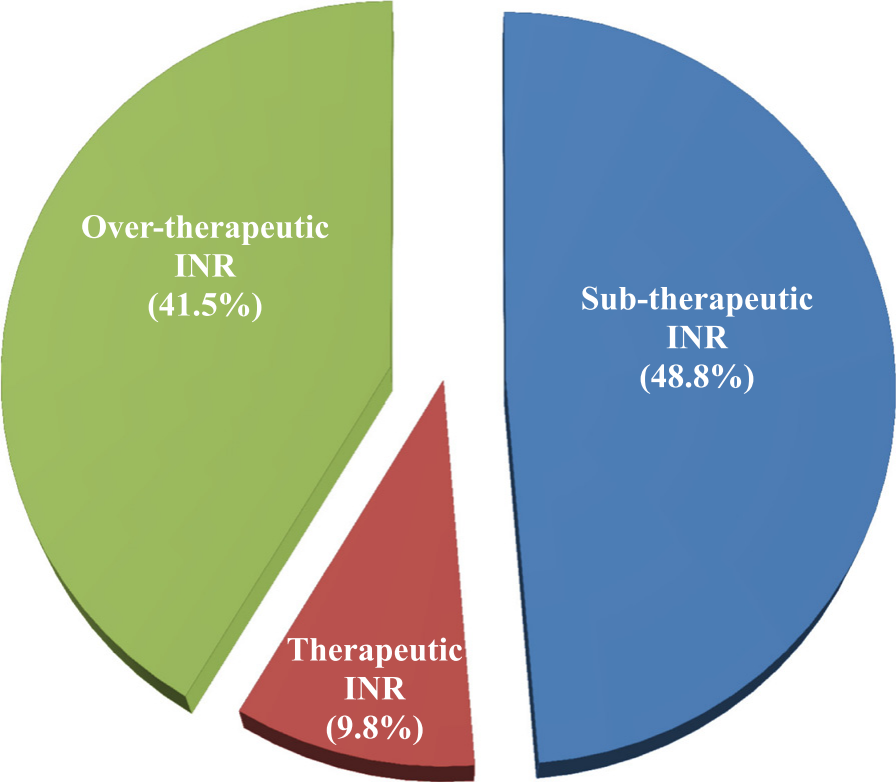
Recent International normalized ratio for which warfarin dose was changed (*n* = 41). Effect of warfarin dose adjustment on the value of INR values was analyzed. Based on recommendations (ACCP 2008), analysis of the effect of warfarin dose was performed for 41 patients for those subsequently documented INR values at least for one week and within two months following the dosage change exists

Effect of warfarin dose on the value of INR values was analyzed based on American College of Chest Physicians (ACCP) guideline [5]. Dose adjustment should be made based on the recent or current INR value and the previous cumulative weekly dose of warfarin. There had to be subsequently documented INR value at least for one week and within two months (56 days) following the dosage change to know the effect of dose. In this study, only 13.4 % (37/276) of nontherapeutic INRs were considered according to the above recommendation. Therefore, analysis of the effect of warfarin dose was performed for 41 patients. Among these, warfarin dosage was adjusted for more than 90 % of non-therapeutic and for about 10 % of therapeutic INRs (Fig. 2).

### Association of warfarin dose and INR values

Adjustment of warfarin dose up or down was done in increments of 16 to 100 % for recent subtherapeutic INRs, 16 to 50 % for therapeutic and 11 to 66 % for overtherapeutic INRs with mean of 36.5 (±18.03) based on the cumulative weekly dose of warfarin. There was a moderate linear relationship between percentage of dosage adjusted and consequent INR values, which was statistically significant (R^2^ = 0.419; *p* = 0.000). This analysis revealed that about 42 % of the variations in INR values were explained by changes in warfarin dosage. Accordingly, 21(51.2 %) of them had a non-therapeutic INR range following dosage adjustment (Table 3).

**Table 3:** Percentage range of warfarin dose adjusted and its effect on the INR values (*n* = 41)

INR values for which warfarin dose was adjusted	*n* (%)	% weekly dose adjusted (mean)	INR after dose adjusted	*n* (%)
Less than 1.5	8 (19.5)	25–50 (37.5)↑	1.5–1.9	2 (25)
			2.0–3.0	5 (62.5)
			>4.0	1 (12.5)
1.5–1.9	12 (29.3)	16–100 (58)↑	1.5–1.9	1 (8.3)
			2.0–3.0	7 (58.3)
			3.1–4.0	2 (16.7)
			>4.0	2 (16.7)
2.0–3.0	4 (9.8)	16–50 (33) *(1 case = 16 %↑; 3 cases = 20-50 %↓)*	< 1.5	1 (25)
			1.5–1.9	2 (50)
			2.0–3.0	1 (25)
3.1–4.0	8 (19.5)	11–33 (22)↓	< 1.5	2 (25)
			1.5–1.9	2 (25)
			2.0–3.0	4 (50)
4.1–5.0	4 (9.8)	16–66 (41)↓	< 1.5	1 (25)
			1.5–1.9	1 (25)
			2.0–3.0	2 (50)
5.1–9.0	5 (12.2)	20–66 (43)**↓**	< 1.5	1 (20)
			1.5–1.9	3 (60)
			2.0–3.0	1 (20)

### Warfarin interactions

#### Drug interaction with warfarin

More than 18 potentially interacting drugs were prescribed concomitantly with warfarin for 38 patients. Among these, 4 drugs were categorized as major interactions which are known to potentiate or inhibit warfarin effect by altering INR and 14 drugs as moderate interaction [6, 7] (Table 4). As a result, more than 55 % (21/38) and 34 % (13/38) of patients were prescribed with drugs that might increase and decrease INR when administered with warfarin, respectively (Table 1).

**Table 4:** Types of drugs concurrently prescribed with warfarin known to have interaction and their potential effect on INR values (*n* = 91)

Drugs	Effect on INR	Severity
Norfloxacin, Metronidazole, Clarithromycin	Increase	Major
Rifampin	Decrease	Major
Isoniazid, Omeprazole, Tramadol, Ceftriaxone, Cimetidine, Allopurinol, Azithromycin	Increase	Moderate
Nevirapine, Neurobion, Mercaptopurine	Decrease	Moderate
Efavirenz, Hydrocortisone, Prednisolone	Increase/Decrease	Moderate
Methotrexate, Vincristine, Furosemide, Simvastatin	Increase	Minor
Hydrochlorothiazide	Decrease	Minor

**Fig. 3:**
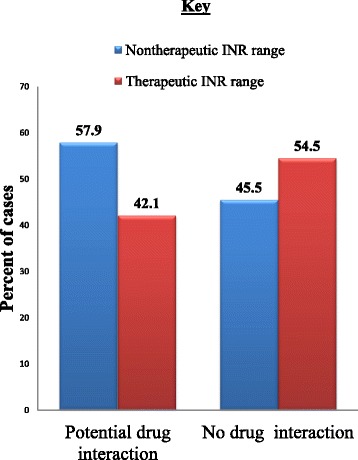
Potential drug interaction at the time of warfarin dose adjustment and consequent INR value (*n* = 41)

Among the patients whose warfarin dosage was adjusted, 46.3 % (19/41) of them were taking other medications during dosage adjustment that would have potential interaction with warfarin. This potential interaction appeared to contribute to fluctuation of INR values, as the values were out of range in a relatively higher proportion of (58 %) patients. Among the 22 patients that potential drug interaction was not a concern, INR was out of range in 45.5 % of the cases. Comparison was made between the two groups that had out-of-range INR to establish the relationship between drug-interaction and INR values. The analysis, however, did not produce any significant difference (OR = 1.65, *p* = 0.43), indicating the lack of association between the two factors (Fig. 3).

#### Food interaction with warfarin

Information about food interaction with warfarin was insufficiently documented except one instruction related to foods containing vitamin K. Thus, more than 12 % (11/91) of patients who were on anticoagulation medication were instructed to avoid green leafy vegetables (rich in vitamin K) during their anticoagulation treatment.

### Adherence to warfarin treatment

Possible causes of non-adherence to anticoagulation treatment were documented in 6.6 % of the studied population and the most common was missing doses (4 of 6 possible causes) and the rest is related to availability of the product. Three of four patients with documented non-adherence had a nontherapeutic INR range.

### ADR associated with warfarin therapy

**Fig. 4:**
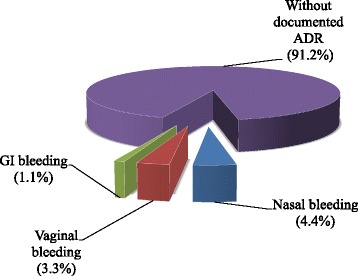
Prevalence of ADR associated with warfarin (*n* = 91)

About 9 % of patients who were on anticoagulation medication were documented to experience minor bleeding events (Fig. 4).

## Discussions

Warfarin dose adjustment was performed for nontherapeutic INR values (more than 66 % of total INR values). Among these, warfarin dose was decreased in 3.4 % of subtherapeutic INRs and increased in 4.2 % of supratherapeutic INR ranges, although there was no clear reason documented for such paradox. In addition, the dose adjustments of only 13.4 % of nontherapeutic INRs were dependent on the recent INR values and cumulative weekly doses of warfarin, as recommended by ACCP guideline [5]. These imply that, most of anticoagulation management might have done by clinical evaluation of the patients’ conditions. Although this approach have paramount importance, it would be much better if supported by available valid guidelines. Such practices might not allow someone to know the direct effect of changes in medication dose and might result in suboptimal quality of anticoagulation management. This could be demonstrated by the low time patients stay in therapeutic INR range (33.5 %). Hence, the percentage of INRs in therapeutic range highly depends on the quality of dose management [5].

Among patients for which analysis of the effect of warfarin dose on the value of INR values was performed, nearly 10 % of INR values for which warfarin dosage had been adjusted were in the therapeutic range. The key informants stated that although dosage changes should be done based on the state of INR, patients’ conditions were taken into account. For example, for patients with certain conditions such as peptic ulcer and other conditions of bleeding events, the risk-benefit ratio would be considered and dosage changes might be done accordingly, even if the INR values were within the therapeutic range. According to other studies, dosage change is not recommended for therapeutic INR values [8], for a single INR that is slightly out of range [9, 10]. ACCP guidelines also do not support dosage change for therapeutic INRs [5]. Therefore, it is important to consider the standard guidelines while deciding to modify/adjust the maintenance anticoagulant doses.

Warfarin weekly dose change ranged between 16 to 100 % for recent subtherapeutic INRs, 16 to 50 % for therapeutic and 11 to 66 % for overtherapeutic INRs. But, according to ACCP guidelines, patients whose INR is just outside the therapeutic range can be managed by either adjusting the dose up or down in increments of 5 to 20 % [5]. Other studies also recommended that dosage changes should be altered by the total weekly dose of 5 to 20 % [8, 9, 11, 12]. Variability in the amount or percentage of warfarin dosage change might be due to the absence of local dosing protocol or failure to adhere to the available international guideline. It is important to adhere to the international standard treatment guidelines where there are no local treatment protocols while deciding to modify/adjust the maintenance anticoagulant dosages for nontherapeutic INR values. Institutional protocols for dosing decisions during maintenance VKA therapy should be developed and used accordingly in order to minimize consequences of DRPs in patients with DVT.

In the present study, change in warfarin dose up or down showed significant linear relationship with consequent INR values (*p* = 0.000). Accordingly, about 42 % of the variations in INR values were accounted for by warfarin dosage change. As a result, percentage of weekly warfarin dosage reduction (up to 70 %) was related to the consequent nontherapeutic low INR values. Therefore, dose reductions might be one of the reasons for low INRs and dosage changes especially higher dose reductions could be major DRPs that might expose the patients to high risk of recurrent thrombosis complications. As evident from the current finding, more than 12 % were presented with significantly under-therapeutic INR range (INR less than 1.5) at weekly warfarin dose reduction of 21–70 %. This finding is in agreement with reports of different studies that, response to a previous change in warfarin dosage was the cause of under-anticoagulation [13] and low INR values [10, 14]. Therefore, high dosage changes greater than 20 % are not advisable and should be avoided in order to minimize complications such as recurrent thrombosis. But, there might be exceptions that need special considerations of higher doses including concurrent medications such as amiodarone and rifampin which need a total weekly dose reduction and increment of 50 %, respectively [15].

Five (12.2 %) patients presented with over-therapeutic INRs at weekly warfarin dosage increment ranging from 11 to 100 %. This might increase the risk of overanticoagulation and thus expose the patients to high risk of bleeding complications as supported by different studies [13, 16]. Dosage increment greater than 20 % should be avoided especially as the dosages become increased more than 40 % the risks might be more. There were 3 cases (7.3 %) in the current study that presented with an INR value greater than 4.0 at greater than 40 % dosage increment. It was not possible to say anything about these patients, as there were no documented adverse events except for some minor undesirable effects (9 %) such as nasal and vaginal bleeding, which were said to be not related to the above factor. But, INR values greater than 3.3 were recorded at the time of these bleeding events although there was no recent dose adjustment done that allows us to correlate with warfarin overdose. Nevertheless, the risk of bleeding should be an issue of utmost importance. Therefore, these trends should be guided by valid treatment guidelines in order to improve clinical outcomes and minimize unwanted complications due to anticoagulant drug overdose.

As the findings of this study showed, there had been potential interactions that could make INR to fall out of the therapeutic range. About 42 % of patients received warfarin concomitantly with 18 other medications that have potentially major or moderate interaction with warfarin. For more than 46 % (19/41) of patients, co-administration was made at the time of dosage adjustment. The fact that about 58 % of patients, in which drug-interaction was a concern, had non-therapeutic INR range suggests that whenever there is a need for dosage changes in patients with DVT the concurrent medications should be considered. It was stated that for most interactions a total weekly dose adjustment of either an increase or decrease of 30 % is needed except for certain drugs such as amiodarone and rifampin which need a total weekly dose reduction and increment of 50 %, respectively [15]. But, in the present study, both dose reductions and increments were done up to 50 % regardless of the type of concurrent medications and this might expose the patients to the risk of recurrent or hemorrhage complications.

Norfloxacin and metronidazole were prescribed for only one and two patients who were on warfarin, respectively. In both cases an increased INR value was showed but no apparent risk of bleeding since the increment was from low to therapeutic INR range. But one study reported that, metronidazole had shown an impact on bleeding risk when used with warfarin concomitantly [17]. In another study, all patients who received quinolone antibiotics concomitantly with warfarin had INR values above 3.5, suggesting drug-induced warfarin potentiation [18]. Rifampin was prescribed for 2 patients. Out of these, one patient’s INR was dropped from 1.31 to 1.27 following warfarin increment of 33 % and the other patient’s INR increased from 1.33 to 2.1 following warfarin increment of 50 %, suggesting major drug-drug interaction and need of large increment of warfarin dose. This is supported by a practice guideline [15], which stated that for rifampin interactions a cumulative weekly dose of warfarin increment of 50 % is needed. Whenever there is a need of anticoagulant dosage changes in patients with DVT, changes in concurrent medications especially those with major interactions with VKAs should be considered.

Tramadol was reported to increase the risk of bleeding [17]. In the present study, this drug was prescribed for 17 patients who were on warfarin therapy. Among these, INR values of 6 patients were increased to supratherapeutic ranges suggesting tramadol-induced warfarin potentiation but difficult to generalize since warfarin dose increment of 20–50 % was performed during this time. In addition, more than 41 % of tramadol prescriptions were written on a PRN (as required) basis without specified maximum daily doses. This might contribute to unintentional administration of over-dosage (more than 400 mg per day) and might also lead to increased effect of warfarin and its bleeding risk. Thus, it would be helpful to write full dosage schedule including the maximum daily dosage whenever medications are ordered especially when prescribed on the basis of PRN.

Vitamin K is known to reverse warfarin’s pharmacologic activity, and many foods containing sufficient vitamin K reduce the anticoagulation effect of warfarin if a patient consumes them in large portions or repetitively within a short period of time. The present study showed that more than 12 % were instructed to avoid green leafy vegetables (rich in vitamin K) during their anticoagulation treatment. Responses from key informants regarding such practice were taken. Some of them suggested that unexplained changes in INR records might force to consider absolute avoidance of diets rich in vitamin K. This was not in agreement with other report that suggested considering abrupt changes rather than avoidance [19]. In the contrary, other informants said this was not the practice and strongly underlined to a consistent and moderate intake of such foods, as also supported by different studies [20, 21]. One study revealed that changes in dietary vitamin K intake were among the causes of INRs below 2.0 and above 4.0 [13]. Others also recommended that patients should be encouraged to maintain consistency in their vitamin K intake and should strive to meet the recommended dietary allowance for vitamin K [19, 21] because dietary content or extent of absorption of vitamin K might have effect on warfarin [16]. Patients should be given a list of vitamin K rich foods and encouraged to maintain consistency in their vitamin K intake rather than absolute avoidance and alternative sources of vitamin K, such as multivitamins and nutritional supplements should also be considered.

It is difficult to assess actual prevalence of patient non-adherence from medical charts. Regardless of this, some possible causes of non-adherence were documented in 6.6 % of studied population and the most common was missing doses. Although three of four patients with documented non-adherence had a nontherapeutic INR range, the low number of these cases compared to patients without non-adherence did not allow us to establish a sound association between non-adherence and INR values. But literature showed that, noncompliance was the cause of under-anticoagulation [13, 14]. Ensuring the patients’ understanding about the benefits of adhering to the prescribed medications and the consequences of non-adherence to treatments is crucial in order to achieve the desired therapeutic goals. Hence patient compliance can influence the frequency of long-term INR monitoring [17].

## Conclusion

In the present study, although young adults were among the commonly affected age groups, VTE is becoming the major cause of concern in all age groups. Managing patients with VTE is more complicated and prone to different DRPs for several reasons, including variable nature of anticoagulants such as warfarin, co morbidities, concurrent medications and other factors related to patients. In this study, different DRPs were identified and the most frequent were sub-therapeutic doses, over-therapeutic doses, and potential drug interactions, highlighting the importance of considering drugs as a possible cause of health problems and the need for their rational use. Warfarin dose adjustment can complicate the treatment course because it demands the knowledge of different factors. Thus, higher dosage changes were among the reasons for non-therapeutic INR values as one of the major DRPs that might expose patients to high risk of recurrent and hemorrhagic complications.

## Abbreviations

ACCP: American College of Chest Physicians
ADR: adverse drug reaction
DRP: drug-related problem
DVT: deep vein thrombosis
FIP: International Pharmaceutical Federation
INR: International Normalized Ratio
OR: odds ratio
PT: prothrombin time
TASH: Tikur Anbessa Specialized Hospital
VKA: vitamin K antagonist
VTE: venous thromboembolism
WHO: World Health Organization
